# Supplementary material for: Plasmodium oocysts respond with dormancy to crowding and nutritional stress
Source: Sci Rep. 2021 Feb 4;11:3090. doi: 10.1038/s41598-021-81574-0 (PMC7862253; doi:10.1038/s41598-021-81574-0)
Supplement: Supplementary file 1 — Supplementary Information 1. [file 41598_2021_81574_MOESM1_ESM.pdf]

Supplementary Information to:

Plasmodium oocysts respond with dormancy to crowding and nutritional stress

Tibebu Habtewold[1]*, Aayushi A. Sharma[1], Claudia A.S. Wyer[1], Ellen K.G. Masters[1], Nikolai Windbichler[1] & George K. Christophides[1]*

[1]Department of Life Sciences, Imperial College London, London, UK

*For correspondence: t.habtewold@imperial.ac.uk, g.christophides@imperial.ac.uk

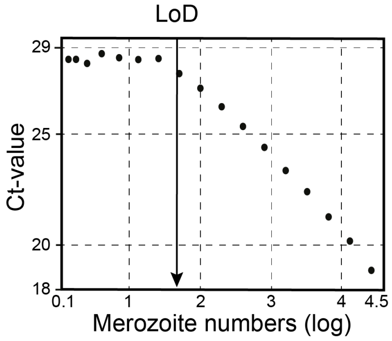

Fig S1. Efficiency of quantitative PCR in determining *PfCyt-b* DNA abundance

Merozoite cells from *P. falciparum* asexual cultures were purified and quantified using FACS sorter. Single midgut homogenates from primiparous *A. coluzzii* mosquitoes were spiked in merozoite serial dilutions. SYBR-based qPCR was performed following DNA extraction. The standards curve represent average of 15 independent qPCR assays. Arrow indicates the limit of assay detection (LoD). Note that each merozoite cell is thought to contain up to 20 mitochondria.

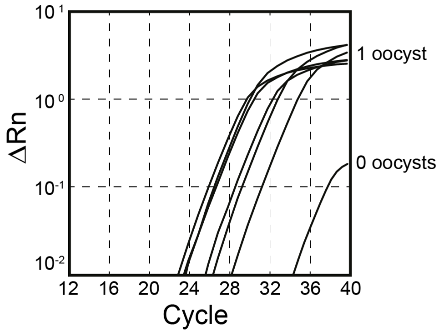

Fig S2. *PfCyt-b* DNA qPCR amplification curves in midguts with single or no oocysts

Midguts of *A. coluzzii* mosquitoes fed on *P. falciparum* gametocytemic blood were dissected at 7 dpi and the number of oocysts were determined. DNA was extracted from 6 midguts showing mono-oocyst infections and 1 with no oocysts. Abundances of *PfCyt-b* DNA in midguts containing a single oocyst in relation to mosquito *S7* gene are 3.24, 6.14, 17.86, 40.37, 0.99, 0.92 and 0.002 from left to right, respectively.

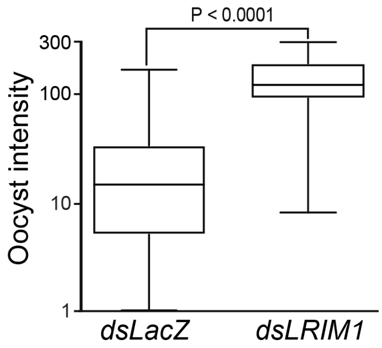

Fig S3. *P. berghei* oocyst intensity following silencing of *LRIM1*

Oocysts present on mosquito midguts were counted at day 14 pi. Injection of *LacZ* dsRNA was used as control. Horizontal lines in boxplots show median. Data were generated from three independent biological replicates. Kolmogorov-Smirnov test was applied to compare oocyst counts between *dsLacZ*-injected and *LRIM1* silenced mosquitoes.

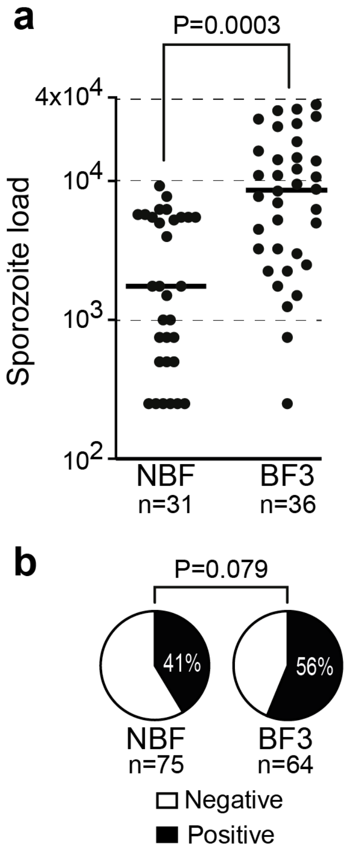

Fig S4. Effect of supplemental bloodmeal deprivation on *P. falciparum* sporozoites

Sporozoite load (**a**) and prevalence (**b**) in mosquitoes on day 15 pi. The mosquitoes were provided no supplemental bloodmeal after the time of infection (NBF) or supplemental bloodmeals 3 dpi. Horizontal lines in dot-plots indicate median.
